# Effect of *Carica papaya* Extract toward Incised Wound Healing Process in Mice (*Mus musculus*) Clinically and Histologically

**DOI:** 10.1155/2019/8306519

**Published:** 2019-11-19

**Authors:** Rachmi Fanani Hakim, Fakhrurrazi, Dinni

**Affiliations:** Faculty of Dentistry, Syiah Kuala University, Jl. Tgk. Hasan Krueng Kalee, Kopelma Darussalam, Kec. Syiah Kuala, Banda Aceh City, Aceh 23111, Indonesia

## Abstract

Wound healing entails a sequence of complex biological processes, which is a protective function of the body that focuses on a quick recovery. Reducing wound healing time is crucial in a wound as it lowers the chance of infection and decreases complications and costs. Papaya extract was obtained by a maceration method. It contains papain enzyme, flavonoid, saponin, and alkaloid, which act as an anti-inflammatory, astringent substance, vasodilator, antioxidant, analgesic, with antifungal, and antibacterial properties, and increase the collagen synthesis. This study aimed to assess the effect of *Carica papaya* extract application toward incised wound healing process in mice (*Mus musculus*) clinically and histologically. This experimental laboratory study was performed using 24 mice weighing between 30 and 40 grams and 12 and 14 weeks of age as experimental animals. Each group was incised along 5 mm at the labial gingiva under both of mandible anterior teeth with a depth reaching alveolar bone. Papaya extract was applied topically in the morning and evening for 14 days. The clinical result was obtained by assessing the length of wound closure measured every day for 14 days. Based on the statistic test result, it showed that the papaya extract has a significant effect (*p* ≤ 0.05) toward the healing process of an incised oral wound in mice. Histologically at day 14, 75% concentration papaya extract showed perfect epithelial layer and fibrillation.

## 1. Background

Wound healing entails a sequence of complex biological processes, which is a protective function of the body that focuses on a quick recovery. Reducing the wound healing time is crucial in a wound as it lowers the chance of infection and decreases complications and costs [1]. Wounds are physical injuries that result in the opening or rupture of tissue, which can cause anatomical and functional disorders. Wounds result in the loss of continuity of the epithelium with or without the loss of underlying connective tissue. Wound healing is a dynamic, complex process that leads to the reestablishment of tissue integrity and homeostasis. It involves inflammation, reepithelization, granulated tissue formation, neovascularization, wound contraction, and remodeling of the extracellular matrix [2].

Surgery is common in the oral cavity. Surgery is performed to correct specific abnormalities and diseases that cause incision wounds in the mucosa involved. Incised wounds occur because mucosa is cut by sharp instruments, for example, cuts in surgery. Clean (aseptic) wounds are usually covered by the suture after all the injured blood vessels have been bound [3].

Oral mucosa wound healing comprises a series of sequential responses that allow the closure of ruptures in tissue involved. This process is of critical importance to prevent the invasion of microorganisms or other agents into tissues, thus avoiding the establishment of chronic inflammation as the oral mucosa is continually exposed to traumatic and infectious challenges [4, 5].

In clinical practice, wound dressing and topical products are used to create and maintain a moist environment and provide adequate conditions for healing [2]. Natural products with medicinal properties can facilitate the wound healing process. Many studies on the wound healing properties of natural products with anti-inflammatory, antioxidant, antibacterial, and pro-collagen synthesis actions have been conducted [6].

The fruit of *C. papaya* is considered as one of the most common fruit related to human consumption and provides a favorable cost benefit in consideration of its nutritional value. *Carica papaya* contains essential nutrients and bioactive compounds, such as antioxidants, vitamins, and minerals. Papaya has the highest concentrations of vitamin C (61.8 mg·100 g^−1^), vitamin A (328 mg·100 g^−1^), riboflavin (0.05 mg·100 g^−1^), folate (38 mg·100 g^−1^), thiamine (0.04 mg·100 g^−1^), niacin (0.34 mg·100 g^−1^), calcium (24 mg·100 g^−1^), iron (0.1 g·100 g^−1^), potassium (257 mg·100 g^−1^), and fiber (0.8 g·100 g^−1^), as well as a low caloric value (32 kcal·100 g^−1^). Terpenoids, alkaloids, flavonoids, and saponins were identified in the water extract as a phytochemical composition of papaya. The healing actions of *C. papaya* are attributed to its several properties. First is papain, its active component, which led to the enzymatic debridement of wounds. The other contributing factor is its vitamin C content, which is essential for the conversion of proline to hydroxyproline, a specific marker, and component of the granulation tissue of the extracellular matrix in wounds [7, 8].

## 2. Methods

This research is an experimental laboratory research (in vivo) using the design of the posttest-only control group and uses grouping based on a completely randomized design (CRD).

### 2.1. Samples

This study uses mice (*Mus musculus*) as experimental animals (male sex, age 12–14 weeks, and weighing 30–40 grams) obtained from the Faculty of Veterinary Medicine, Syiah Kuala University. The number of mice that were used is 24 heads divided into four groups, and each group consisted of six mice. The repetition was done as much as the treatment.

### 2.2. Papaya Extraction

The papaya extract was made by the maceration method. The maceration process was carried out once. The results of this filtering were further concentrated using a vacuum rotary evaporator at 40°C to produce 100% papaya fruit extract. The 100% concentration then was diluted to a concentration of 25%, 50%, and 75% according to the concentration dilution formula. Ten milliliters of distilled water were added to 2.5 grams, 5 grams, and 10 grams of papaya extract to produce a concentration of 25% (P1), 50% (P2), and 75% (P3), respectively.

### 2.3. Treatment of Mice

Experimental animal injuries were performed on the labial gingiva under the anterior teeth of the mouse mandible. Beforehand, mice were anesthetized using topical lidocaine ointment. The injury was carried out using a scalpel and blade no. 15 along 5 mm with a depth reaching the alveolar bone. This study used four treatments, namely, the control group negative (K0), treatment group I (P1), treatment II (P2), and treatment III (P3). The negative control group was treated with distilled water, the first group (P1) was treated with a concentration of 25% of papaya extract, the second group (P2) was treated with a concentration of 50% of papaya extract, and the third group (P3) was treated with a concentration of 75% of papaya extract. Each sample group was treated with the same wound, which was twice a day using a disposable microapplicator. The topical papaya extracts were applied twice a day with the same concentration.

### 2.4. Wound Care and Observation

Each group of samples was treated for the same wound, which is twice a day at 08 : 00 AM and 08 : 00 PM West Indonesia Time. Wound care began on the first day of injury. During wound care, operators washed their hands and used sterile gloves. Observations were made clinically by assessing the length of wound closure measured every day for 14 days using periodontal probe (UNC-15). Observations of this study were carried out macroscopically and microscopically.

### 2.5. Data Analysis

The data were analyzed using a one-way ANOVA test with 95% confidence interval (*α* = 0.05). The analysis was continued with Duncan's test to see the extract concentration that was most influential on wound healing.

## 3. Results

### 3.1. Clinical Wound Healing Observation

The results of the application of papaya extract concentrations of 25%, 50%, and 75%, as well as distilled water, showed healing that was marked by a reduction in wound length. Measurement of wound length is done clinically by assessing the length of wound closure measured every day for 14 days using periodontal probe (UNC-15). The results of the measurement of the wound length of each treatment group can be seen in Table 1.

The results of this study indicated that the wound length generally began to decrease on the fourth day; even though in the treatment group III (P3) papaya extract was applied with a 75% concentration, there was a reduction in the wound length on the third day. Observations were made up to the 14th day in each group of 24 mice that were sampled as many as 7 mice.

In the negative control group (K0), which was applied with distilled water, wound healing only occurs as long as 1–2 mm from the wound. The wound length began to decrease on the eighth day, but two mice (Nos. III and IV) had a reduction in the wound length on the fourth day and seventh day, respectively. Mouse No. VI in the negative control group died after injury.

In the treatment group I (P1), which was applied with 25% of papaya extract, wound healing occurs along 2–3 mm of injury. In this group, the length of the wound began to reduce on the fourth day. In general, the picture of healing in this group looked almost the same.

In the treatment group II (P2), which was applied with 50% of papaya extract, wound healing occurred 3–5 mm from the wound. In this group, the length of the wound began to decrease on the fourth day. On the same day, it was seen that one mouse (No. II) experienced 2 mm of healing. On the 14th day, one mouse (No. VI) experienced healing which was marked by a reduction in wound length to 0 mm.

**Figure 1:**
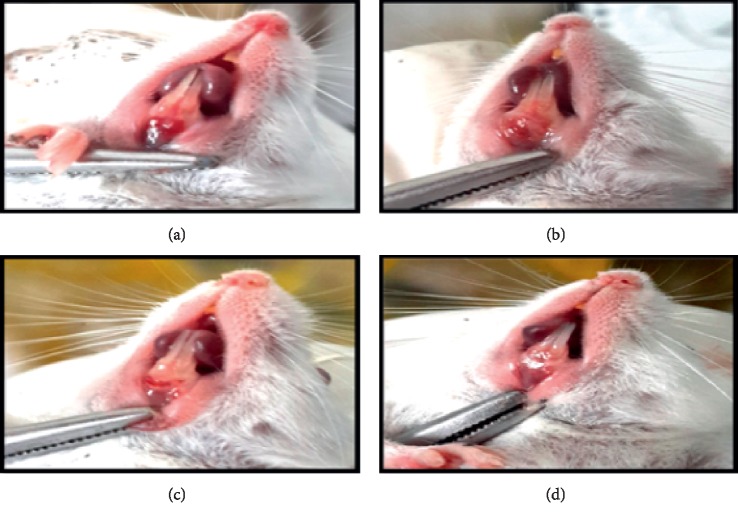
Comparison of wound healing on day 14: (a) negative control group, (b) treatment group I, (c) treatment group II, and (d) treatment group III.

**Figure 2:**
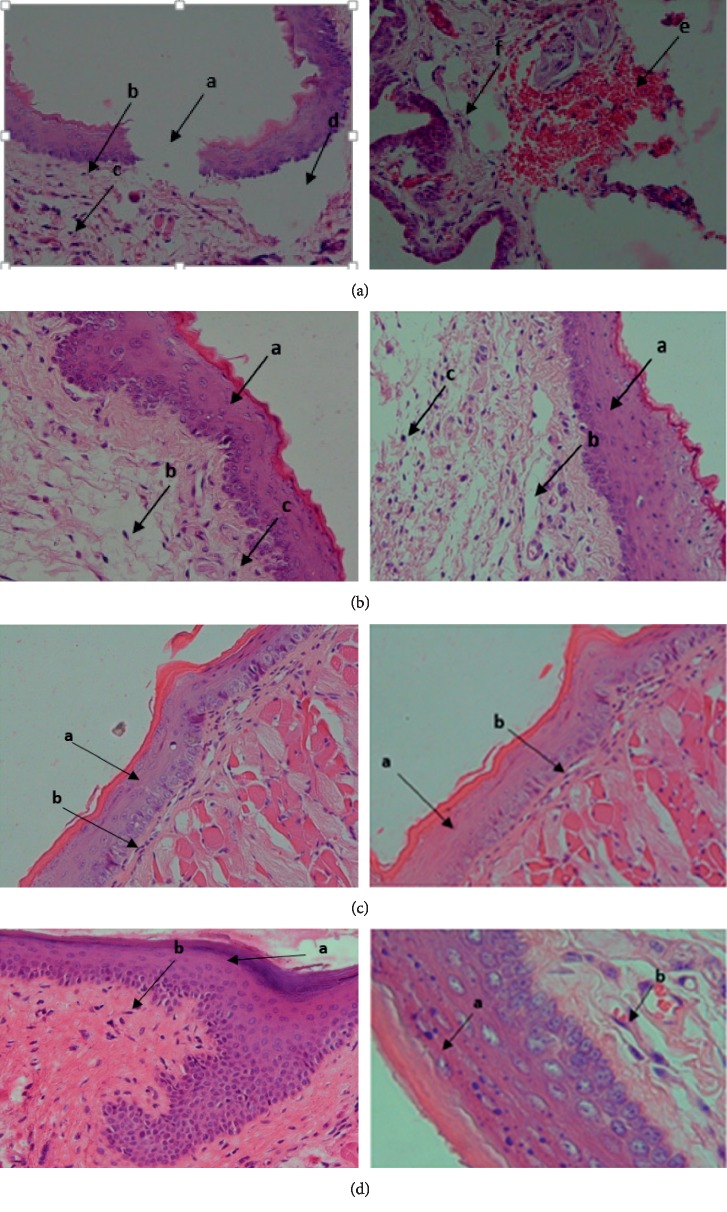
(a) Histological view of negative control on day 14. (A) Epithelial layer is still separated. (B) Slight fibers and not tight. (C) Inflammation of inflammatory cells. (D) The existence of areas where fibrillation did not occur. (C) Hemorrhage. (F) Inflammatory cells (HE, 10 × 40). (b) Histological overview treatment group I. (A) The composition of the epithelium is perfect. (B) Fibroblast are sparse, thin, and poorly arranged. (C) Inflammatory cells (HE, 10 × 40). (c) Histological Overview Treatment group II. (A) Perfect epithelial layer. (B) Fibrillation densely and neatly arranged (HE, 10 × 40). (d) Histological Overview Treatment group III. (A) Perfect epithelial layer. (B) Perfect fibrillation (HE, 10 × 40 and 10 × 100).

In the treatment group III (P3), which was applied with 75% of papaya extract, wound healing occurred along 5 mm. In this group, the length of the wound began to decrease on the third day. Healing went very well in this group compared to other groups. On the 11th day, there was a cure in 50% of the mice tested and the rest experienced healing on the 12th day (Figures 1 and 2).

### 3.2. Histological Observation

The results of the histologic feature can be seen in Figure 2.

## 4. Discussion

Wound healing is a process of the body replacing devitalized and missing tissue in order to fill the lost and repair the damaged. Usually, wounds are healed by primary or secondary intention. It depends on whether the wound is covered with sutures or left to repair. Where the formation of connective tissue restores the injured tissue, regrowth of the epithelium is seen [9]. In the granulation tissue, fibroblasts are activated and play a vital role in supporting normal wound healing, involved in key processes such as breaking down the fibrin clot, creating new extracellular matrix (ECM) and collagen structures to support the other cells associated with effective wound healing, and contracting the wound [10].

Many researchers that investigated wound healing found that topical administration of herbal gel formulation significantly enhances wound healing in rats. Roodbordei et al. investigated topical hydrogel containing *Fumaria vaillantii* Loisel. extract enhances wound healing in rats [11]. The topical effect of grape seed extract 2% cream on surgery wound improves cellular structure and wound contraction as investigated by Hemmati et al. [1]. Magalhães et al. investigated effect of a combination of medium-chain triglycerides, linoleic acid, soy lecithin, and vitamins A and E on wound healing in rats, and the results did not accelerate the process of tissue repair by secondary union [12]. Mi Hyun Kang and Bae-Hwan Kim found that water extract of acai berry also had oral wound healing effect both clinically and histologically [13].

In this research, *Carica papaya* extract can increase epithelial thickness and fibrillation. The length of the wound began to decrease on the third day. The application of papaya extract showed an increase in the number of wound lengths that healed compared to the negative control group that applied aqua destilata. Papaya extract was assessed for the first time on healing of the incised wound in mice (*Mus musculus*) with successful results. So, it can be concluded that *Carica papaya* extract had a significant effect on epithelization and fibrillation. Also, the time of wound contraction was significant in this group that can be recommended in the healing of oral surgical wounds. 75% concentration papaya extract showed perfect epithelial layer, fibroplasia, and wound contraction.

## Figures and Tables

**Table 1:** Measurement of wound length (mm).

Group		Day													
		1	2	3	4	5	6	7	8	9	10	11	12	13	14
Negative control group	I	5	5	5	5	5	5	5	4	4	4	4	4	4	4
	II	5	5	5	5	5	5	5	4	4	4	4	4	4	3
	III	5	5	5	4	4	4	4	4	4	4	4	4	4	4
	IV	5	5	5	5	5	5	4	4	4	4	4	4	4	3
	V	5	5	5	5	5	5	5	4	4	4	4	4	4	4
	VI														

Treatment group I (25% papaya extract)	I	5	5	5	4	4	4	4	3	3	3	2	2	2	2
	II	5	5	5	4	4	4	4	3	3	3	2	2	2	2
	III	5	5	5	4	4	4	4	4	3	3	3	3	3	3
	IV	5	5	5	4	4	4	4	3	3	3	2	2	2	2
	V	5	5	5	4	4	4	4	4	3	3	3	3	3	3
	VI	5	5	5	4	4	4	4	4	3	3	3	3	3	3

Treatment group II (50% papaya extract)	I	5	5	5	4	4	4	3	3	3	2	2	1	1	1
	II	5	5	5	3	3	3	3	3	2	2	2	2	2	2
	III	5	5	5	4	4	4	4	3	3	3	3	2	2	2
	IV	5	5	5	4	4	4	4	3	3	3	2	2	2	2
	V	5	5	5	4	4	3	3	3	3	2	2	1	1	1
	VI	5	5	5	4	4	3	3	3	2	2	2	1	1	0

Treatment group III (75% papaya extract)	I	5	5	5	4	4	3	3	2	1	1	0	0	0	0
	II	5	5	4	3	3	3	2	2	1	1	1	0	0	0
	III	5	5	5	4	3	3	2	2	1	1	0	0	0	0
	IV	5	5	5	4	4	3	3	2	1	1	0	0	0	0
	V	5	5	4	3	3	3	2	2	1	1	1	0	0	0
	VI	5	5	4	3	3	3	2	2	1	1	1	0	0	0

## Data Availability

The data that support the findings of this study are available from the corresponding author.
